# Infection Control Knowledge, Attitudes, and Practices among Students of Public Dental Schools in Egypt

**DOI:** 10.3390/ijerph18126248

**Published:** 2021-06-09

**Authors:** Christina El-saaidi, Omid Dadras, Patou Masika Musumari, Masako Ono-Kihara, Masahiro Kihara

**Affiliations:** 1 International Institute of Socio-Epidemiology, Kyoto 606-8336, Japan; patoumus@yahoo.fr (P.M.M.); kihara.masako.0612@gmail.com (M.O.-K.); kihara.masahiro.44z@st.kyoto-u.ac.jp (M.K.); 2 Unit for Global Health, Centre for the Promotion of Interdisciplinary Education and Research, Kyoto University, Yoshida Hon-Machi, Sakyo-ku, Kyoto 606-8501, Japan; 3 School of Public Health, Walailak University, Nakhon Si Thammarat 80160, Thailand; omiddadras@yahoo.com

**Keywords:** infection control, dental schools, needlestick

## Abstract

In developing countries such as Egypt, the risk of blood-borne diseases such as human immunodeficiency virus, hepatitis B virus, and hepatitis C virus is high for healthcare workers. To evaluate infection control knowledge, attitudes and practices, as well as the associated risk of percutaneous infection among dental students, a cross-sectional study was conducted in four Egyptian public dental schools in 2016. A total of 1776 students received an anonymous questionnaire on infection control knowledge, attitudes, and practices and the occurrence of needle and sharps injuries; 1067 (60.1%) completed the questionnaire. Third- (pre-clinical), fourth- (junior-clinical), and fifth-year (senior-clinical) students comprised 44.2%, 15.6%, and 40.2%, respectively. Although the majority of the students reported good attitudes and practices for infection control, knowledge scores were generally low. Female students scored higher on self-protection and sterilization practices than did male students, and the fourth-year students showed significantly higher scores for infection control practice than did the fifth-year students. In multivariate analysis, higher scores for all infection control practices were associated with higher scores for attitudes towards infection control and fewer (1–3) needle injury experiences. Although an alarming proportion had experienced needle or sharps injuries during clinical training, around 30% of the students had not received a complete hepatitis B vaccination. Future infection control education should introduce refresher training before graduation that focuses on injury prevention and post-exposure protocols. Additionally, introducing safer products and clinical procedures is highly recommended to minimize the risk of injuries during clinical practice for dental students in Egypt.

## 1. Introduction

Blood-borne pathogens transmitted through bodily fluids, such as human immunodeficiency virus (HIV), hepatitis B virus (HBV), and hepatitis C virus (HCV), are a major health concern [1,2]. Healthcare providers are at three to six times greater risk of blood-borne infections than other populations [3,4,5]. Healthcare providers in developing countries are also at a higher risk. According to a 2002 World Health Organization (WHO) report, hepatitis B and C account for 40% and HIV accounts for 2.5% of the global burden of disease attributable to occupational exposure among healthcare workers [6], with 90% of such exposures occurring in developing countries [7]. Regionally, a report by the WHO Eastern Mediterranean Regional Office estimated that around 1.4 million people acquire HBV and HCV infections during healthcare delivery in the Middle East [8]. Of these countries, Egypt is facing an alarming epidemic of blood-borne infectious diseases, particularly HCV, which is among the highest in the world. A study conducted in Egypt in 2013 showed that the prevalence of HCV was as high as 55% in some populations [9], and HBV prevalence reached 18.5% in the general population, according to a 2015 DHS report [10]. Although Egypt has made considerable progress in fighting HCV infections through a national control strategy including testing and treatment [11], sources of infections at the community level are still vague and difficult to tackle. The reported HIV prevalence is estimated to be low (0.1%); however, many factors hinder HIV surveillance and reporting in Egypt, which might underrepresent the situation [12].

Dental care providers are at particular risk since treatment involves intrusive procedures with a high possibility of contamination by microorganisms from blood or saliva [5,13,14,15,16]. The risk of infection in dental care could be the result of a lack of HBV vaccination and poor adherence to universal precautions, which emphasize that “all blood and blood-contaminated fluids are potentially infectious” [17,18]. Studies from developing countries have shown that adherence rates to infection control measures in dental practice were much lower than in developed countries [19,20,21]. Although universities are the basic source of infection control education for future dentists, research in Middle Eastern countries has found that knowledge of infection control is low among dental students and even some educating staff [22,23,24,25,26,27]. Research addressing this issue in Egypt is scarce and has not covered the full range of infection control knowledge, attitudes, and practices among students. Therefore, this study assessed these factors and the possible correlates of infection control practices among dental students in Egypt, including their possible exposure to needle and sharps injuries during clinical training, as well as the coverage of the HBV vaccination program. We hypothesized that inappropriate infection control practices may be associated with poor attitudes toward infection control and therefore, greater exposure to needle and sharps injuries during clinical training among students. We targeted public rather than private dental schools in Egypt because they have more students and graduates. Additionally, public dental schools’ clinics provide dental care services for broader low-income populations, including the rural population who are at greater risk of infectious diseases, particularly HCV [28,29].

## 2. Materials and Methods

### 2.1. Study Setting and Sample Selection

A cross-sectional study was conducted among third- (pre-clinical trainee), fourth- (junior-clinical trainee), and fifth-year (senior-clinical trainee) students who had begun clinical training at the four largest public dental schools in Egypt between February and April 2016. Through a primary investigation, the classes with the highest attendance rates were identified at each school. Of the 4223 formally registered students, through a convenience sampling approach, 1776 students could be reached by the interviewers at the end of lectures. This was mainly due to the low attendance rate of dental students in Egypt because private courses outside the university are preferred. After explanation of the study objectives and obtaining verbal consent, an anonymous questionnaire was delivered, and 1067 participants answered and returned it by the end of the survey.

### 2.2. Measurement and Instrument

A self-administered questionnaire was adapted from the main investigator’s previous study on infection control at dental clinics in Yemen [20]. The questionnaire, except for medical terms and the name of the instrument, was in Arabic, the official language of Egypt. A pilot study, recruiting 40 students (20 from the third year and 20 from the fourth and fifth years) at one school, was conducted to evaluate the validity and reliability of the questionnaire. These participants were excluded from the main survey. Face validity was assessed by checking the appropriateness of the translation and relevance of the questions. Sample questionnaires were distributed to the pilot sample and re-tested 12 days later. Questions with a kappa coefficient of 0.7 or higher were included in the final questionnaire. Although no question was excluded due to a low kappa coefficient, some were removed because of irrelevance or the difficulty of answering. Finally, two versions of the questionnaire were developed: one for third-year students without questions on infection control practices and one for fourth- and fifth-year students with questions on infection control practices. Attitude and knowledge questions were included in both types of the questionnaire. The final questionnaire comprised 65 main questions and four sub-questions divided into the following six domains:

(1) Demographic and academic background: Four questions on gender, age, year grade, and whether dentistry education was obtained from the programs other than the school where they were registered.
(2) Infection control practices: Four parts with 22 questions each, using five Likert-scale options of “never”, “rarely”, “sometimes”, “often”, and “always”. These gave a score from 0 to 4 in this order, except for some negative questions coded conversely. This domain consisted of four subdomains:
  - Self-protection: Eight questions assessing basic self-protection measures during clinical practice, including barrier protections such as gloves and eye protection and hand hygiene (total score = 0–32).
  - Sterilization: Nine questions assessing sterilization of surgical tools and re-use of disposables such as anesthesia needles and cartridges during training (total score = 0–36).
  - Injury management: Three questions assessing appropriate reporting and management of accidental cutaneous injuries with needles or sharps (total score = 0–12).
  - Waste management: Two questions assessing whether sharps and biological waste were disposed of properly (total score = 0–8).
(3) Attitude: This domain comprised three parts:
  - Risk perception: Three questions assessing risk perception for blood-borne infectious diseases (HBV, HCV, and HIV) in dental practice with four Likert-scale points of “never expected”, “not very much expected”, “expected”, and “highly expected”. These gave a score from 0–3 in this order (total score = 0–9).
  - Attitude toward patients: Three questions on patients with HBV, HCV, or HIV, with four answer options of “I would not treat the patient”, “a lot of concern but I would treat”, “I would treat with some concern”, and “I treat”. These gave a score of 0–3 in this order (total score = 0–9). Each question was followed by a sub-question to reveal possible factors for rejecting the treatment of the patient.
  - Attitude toward infection control: 12 questions assessing attitude toward infection control measures in dental practice with five Likert-scale points of “absolutely disagree”, “somewhat disagree”, “neutral”, “agree”, and “strongly agree”. These gave a score of 0–4 in this order (total score = 0–48), except for some negative questions coded conversely.
(4) Knowledge: This domain comprised two parts:
  - Infectious disease knowledge: Six questions assessing the level of knowledge on blood-borne infectious diseases (HBV, HCV, and HIV) in dental practice; three on transmission routes (blood, unprotected sex, and saliva) and three on prevention measures including vaccination and precautions. Each question included six answer options, of which 1–3 were correct depending on the question. The correct answer was given one point, with the total score ranging from 0–6.
  - Infection control measures: 11 questions regarding basic and crucial infection control measures according to the guidelines of the US Centers for Disease Control and Prevention for the dental practice. Four answer options were possible, of which only one was correct. The correct answer was given one point, with the total score ranging from 0–11.
(5) Experience of injury: Two questions on injury within the last 6 months, one addressing needlestick and the other injury by sharp instruments during treatment. Each had five answer options: “always”, “often (4–5 times)”, “sometimes (2–3 times)”, “rarely (one time)”, and “never”.
(6) HBV vaccination: Two questions, one on HBV vaccination (yes/no) and the other on the number of doses received within 6 months (“1”, “2”, “3”, “currently receiving doses”).

Since no documented “gold standard” exists to define proper practice or poor practice, we considered the relativity and comparison between groups based on the median score. Scores above the median indicated better practice, attitudes, and knowledge; scores below the median were considered poor (Questionnaire S1).

### 2.3. Data Collection and Management

After a brief introduction to the study and obtaining informed consent to participate, the questionnaire was distributed by six assistants among students at the end of lectures. They were asked to complete the questionnaire and leave it at the front desk of the lecture hall to ensure anonymity. The questionnaires were collected at the end of the session, and the data were extracted and entered into an Excel spreadsheet by the main investigator for analysis. For the practice, attitude, and knowledge questions, answers with missing responses to more than half of the items were treated as missing data. Missing values were imputed with the mean score of valid responses for the questions with valid responses in half or more of the items (within-subjects imputation) [30]. Missing value imputation was applied in 19–62 cases, depending on the question (Data S1).

### 2.4. Statistical Analysis

After data validation and cleaning, statistical analysis was performed using SPSS for Windows (Version 21.0; SPSS Inc., Chicago, IL, USA). Univariate analysis was performed to calculate the mean, standard deviation, and median of the scores. Comparisons of score means between year groups and genders were performed using Student’s *t*-test or one-way analysis of variance (ANOVA). When the ANOVA result was statistically significant, a Scheffé post hoc test was employed to determine the differences between the two groups. Categorical variables were compared with a Chi-square test, and wherever 20% of the expected cell counts was less than 5, Fisher’s exact test was employed. To examine the association between each outcome variable (self-protection, sterilization, injury management, and waste management) and independent variables (gender, year group, HBV vaccination status, injury experience, risk perception, attitudes, and knowledge), the outcome variables and some of the independent variables were dichotomized as above or below the median. The HBV vaccination variable was categorized into “complete vaccination course”, “incomplete vaccination course”, and “not vaccinated”. Needle and sharps injury experiences were both categorized into “never”, “1–3 times”, and “>3 times”.

Before multivariate analysis, bivariate binary logistic regression analyses were performed between each outcome variable and independent variable. Only independent variables associated with each outcome variable with *p* < 0.15 were entered in the multivariate logistic regression analysis after confirming the absence of multicollinearity. The results were reported as odds ratio (OR) and 95% confidence interval (95% CI). A *p*-value less than 0.05 was considered statistically significant.

## 3. Ethical Considerations

This study was conducted according to the International Guidelines for Ethical Examinations of Epidemiological Study (CIOMS, 1991; Geneva, Switzerland) and Declaration of Helsinki principles. Ethical approval was obtained from the Kyoto University Graduate School of Medicine Ethical Committee (Ethical Approval No. R00063). Additionally, approval was obtained from the dean and student affairs office of each participating dental school. The objectives of the study and questionnaire content were thoroughly explained to the students, and they were informed of their right to refuse or withdraw participation at any point. Answering and returning the questionnaire was considered informed consent. Anonymity was ensured by eliminating any personal information from the questionnaire.

## 4. Results

### 4.1. Distribution of the Participants

Among 1776 eligible and available students, 1067 (60.1%) answered and returned the questionnaires, 329 (30.8%) were males and 738 (69.2%) were females. The number of 3rd-, 4th-, and 5th-year students were 472 (44.2%), 166 (15.6%), and 429 (40.2%), respectively. The age group of 19–22 years, 22–24 years, and over 24 years represented 526 (49.3%), 536 (50.2%), and 5 (0.5%), respectively. Of all eligible and available students 217, 164, 279, and 407 represented the students from four public dental schools.

### 4.2. Infection Control Practices

As indicated in Table 1, the students generally showed good scores for infection control practices, with the mean or median being 80–100% of the full score, except for injury management, which was 65–80% of the full score. Fourth-year students showed significantly higher mean scores for infection control practices than those in the fifth year (self-protection: 27.9 vs. 26.6, *p* < 0.001; sterilization: 32.6 vs. 31.5, *p* < 0.001; injury management: 8.9 vs. 7.7, *p* < 0.001; waste management: 6.7 vs. 6.2, *p* = 0.001). The mean score for self-protection was significantly higher in females than males in the fourth year (28.3 vs. 27.0, *p* = 0.005) and the fifth year (26.9 vs. 25.9, *p* = 0.008). Mean scores for sterilization were also higher among female students in both year groups (4th year: 32.9 vs. 32.1; 5th year: 31.9 vs. 30.2); however, the difference was significant only in the fifth year (*p* < 0.001). In contrast, mean scores were lower in female than male students for injury and waste management, without statistical significance in both year groups, except for injury management in the fourth year, which was significantly higher in female students than in male students (*p* < 0.001).

### 4.3. Infectious Diseases and Infection Control Risk Perception, Knowledge, and Attitudes

As Table 2 shows, the mean scores for risk perception and attitudes towards infection control ranged from 55–67% and 85–92% of the full scores, respectively. The scores for attitudes towards patients and knowledge of infectious diseases and infection control were low, at less than 50% of the full scores. The differences between years were statistically significant for all questions, with a general trend of mean scores higher in the fourth- and fifth-year students compared to the third-year students. However, the risk perception score was highest in the third-year students. No gender difference was found in the fourth-year students for any of the domains. However, a significant difference was observed between genders for the attitudes towards infection control scores and patients in the third- (*p* < 0.001) and fifth-year students (*p* = 0.001). For the knowledge domains, a gender difference was observed in the knowledge of infectious diseases in the third-year students and in the knowledge of infection control in the fifth-year students.

### 4.4. HBV Vaccination and Needle and Sharps Injury Experience

As shown in Table 3, around 30% of the students either were not vaccinated or had received an incomplete HBV vaccination course. Around 60% and 38% had experienced needle and sharps injuries during the last 6 months, respectively. Although no statistical difference was observed in the HBV vaccination status between years, the fifth-year students had experienced significantly more needle and sharps injuries than the fourth-year students (needle injuries: 64.3% vs. 43.6%, *p* < 0.001; sharps injuries: 42.3% vs. 27.1%, *p* = 0.004). While more than 70% of the female students had received a complete HBV vaccination course, this was only 54–63% in the male students, with the gender difference statistically significant in both the fourth-year (*p* = 0.017) and fifth-year students (*p* = 0.006). Female students had experienced more needle and sharps injuries than had male students (needle injuries: 52.0% vs. 26.9%, *p* = 0.012; sharps injuries: 31.1% vs. 19.3%, *p* = 0.131) in the fourth year. Furthermore, in the fifth year, female students tended to have more sharps injury experience than did male students (45.4% vs. 33.9%, *p* = 0.08). No statistically significant difference was observed in needle injuries between genders.

### 4.5. Relationship between Needle or Sharps Injury and HBV Vaccination Status

Students who had received a complete vaccination course were more likely to have experienced needle or sharps injuries compared to those who had not. However, this difference was not statistically significant. This tendency was observed only in males in both the fourth- and fifth-year students, except for the fourth-year female students for needle injuries (Table S1).

### 4.6. Bivariate and Multivariate Analysis for Correlates of Infection Control Practices

In the bivariate logistic regression analysis (Table 4), a higher score (≥median) for infection control practice in all domains (self-protection, sterilization, injury management, and waste management) was significantly associated with being in the fourth year (crude odds ratio [COR] ranges 1.7–2.2) and a higher score (≥median) for attitude towards infection control (COR = 1.9–3.1). Female students scored better in self-protection (COR = 1.6) and sterilization (COR = 1.8); however, they scored lower in waste management (COR = 0.7) compared to male students. Never having experienced needle injuries was associated with higher scores for self-protection (COR = 2.1), sterilization (COR = 3.1), and injury management (COR = 3.1). Never having experienced sharps injuries was only associated with sterilization (COR = 2.5) and injury management (COR = 2.0).

A similar profile for associated risk factors was observed in the multivariate logistic regression analysis; however, the association between never having experienced needle injuries and self-protection, and the association between never having experienced sharps injuries and sterilization and injury management, were weakened and lost statistical significance after adjustment for other variables (Table 4). 

## 5. Discussion

This study is the first survey to address practices, attitudes, and knowledge related to infection control, occupational injuries, and HBV vaccination in major public dental schools in Egypt. Although most participants had high scores for infection control practices and attitudes, the knowledge of infectious diseases and infection control was low. The infection control practices were significantly better in fourth-year than fifth-year clinical students. Furthermore, the fifth-year students were more likely to have experienced needle or sharps injuries despite their greater clinical experience, and female students showed significantly better scores for self-protection and instrument sterilization than did male students. Among infection control practices, injury management had the lowest score. Although approximately half of the students had experienced needlestick and sharps injuries, a substantial proportion were not fully aware of post-exposure preventive measures. Moreover, only a third of students, predominantly female, had received the full course of HBV vaccination within the last 6 months.

Although knowledge scores for infectious diseases and infection control were generally low among our study participants, the scores were not associated with any type of infection control practices. This might suggest that infection control practices are unlikely to be supported as part of the basic knowledge of infectious diseases and infection control measures provided at the beginning of clinical training [31]. It might also explain the declining infection control practices and risk perception over the year groups [32]. Therefore, effective hands-on infection control education and training that encourages adherence to such measures are necessary.

Fourth-year students scored better in infection control knowledge, attitudes, and practices than did fifth-year students; similar findings have been documented in studies from other developing countries [33,34,35]. This trend suggests that while getting used to clinical practice, senior students may relax their attention to infection control [36]. Additionally, our results showed low risk perception scores in the third- and fourth-year students and even lower scores among the fifth-year students. This finding of the lowest infection control practice scores in the fifth year reinforces the notion that these students relax their attention to infection control protocols. This could also explain the higher incidence of injuries in senior students compared to junior students in the last 6 months. Therefore, students should be re-educated and trained in infection control before graduation.

Previous studies have shown that female students and health workers are more cautious in practicing infection control protocols [37]. Likewise, in our study, female students scored significantly better for self-protection and instrument sterilization, although no gender difference was observed in injury or waste management practice. In addition, the proportion of female students who had completed HBV vaccination was much higher than for male students, and they were more likely to report needle and sharps injuries. Similar trends have been observed among dental students in other countries [38,39]. This may be due to the tendency of female students to recall such incidences more accurately than male students [40,41]. Otherwise, female students might be exposed to more injury incidences during clinical practice [39,40,41]. More research is needed to determine whether the reporting rate or risk of exposure is more likely, to provide female students with more effective protection from needlestick and sharps injuries. Additionally, the fifth-year students experienced significantly higher needle and sharps injuries than the fourth-year students, which has also been reported in other studies [37]. This might be explained by the higher number of patients senior-level students encounter in the clinical training, as well as the more complex clinical cases that require the use of sharp instruments and needles more frequently, especially in oral surgeries. Generally, around 60% and 40% of the students had experienced needle and sharps injuries, respectively, which has been also documented in other developing countries such as Yemen, Iran, and Nigeria [37,38,41,42]. This highlights the critical need for post-exposure risk management among dental students.

Students scored the lowest in injury management, and a substantial proportion of students were not fully aware of post-exposure preventive measures. Moreover, around half of the students scored low in injury management and had experienced such injuries more than three times within 6 months.

In this study, around 30% of the students had either not received or not completed an HBV vaccination course and thus were more vulnerable to blood-borne infections. Nevertheless, even students who are vaccinated for HBV could still be at risk of other blood-borne diseases such as HIV and HCV. Hence, given the epidemic of HBV and HCV in Egypt, protecting students from blood-borne infections should require not only compulsory HBV vaccination for all students before practice but also rigorous training on needle and sharps injuries.

Dental students continue to experience occupational injuries in clinical practice, even after graduation [43]. Given the negative association of needle and sharps injury incidence with infection control practice scores observed in our study, continuous education on injury management should be introduced for both undergraduate and graduate students, with continuous monitoring to ensure the safety of students during clinical practice.

The attitude toward patients with infectious diseases was generally poor among participants, showing a reluctance to treat patients with infectious diseases. This finding is common in not only developing but also developed countries; however, developed countries have shown better attitudes [23,44,45,46,47]. Therefore, reducing the incidence by implementing rigorous safety measures and enhancing confidence in them during clinical practice at dental schools is important. Ethical responsibility towards patients with infectious diseases should also be promoted in students [47].

Finally, though our study was conducted only among four dental schools in Egypt, the implication of our results brings a broader view based on the public health situation in Egypt. This is because dental students who are already at substantial risk of blood-borne infections during clinical training will be probably more so in clinical practice after graduation. Moreover, if they get infected, it could contribute to the transmission chain of these infections in society. The fact that our study was conducted in the largest, top-ranked dental schools with the highest number of students in the country would intensify this notion. The situation of knowledge, attitude, and practices for infection control among dental students revealed in our study is likely to be shared with dental education environments under the same or more limited resources in other developing countries.

## 6. Strengths and Limitations

Our study is the first to cover a wide range of public dental schools in different cities and settings around Egypt. We developed a unified tested questionnaire that could be used in different schools and can be adapted for future research in other developing countries. However, the study faced several limitations. First, our results reflect only one-quarter of the registered students at the four participating schools. This potentially affects the representativeness of the sample. However, the sampling was in an environment where attendance is not a prerequisite for final academic evaluation, with an emphasis on voluntary participation in the survey. Second, we imputed missing values for a proportion of the questionnaires, which may have introduced bias. However, this was unlikely because the results remained almost the same after eliminating all cases with missing values from the analyses. Third, our results may have been influenced by recall bias for the questions on experiences of needle or sharps injuries and vaccination, although we limited the timeframe to the previous 6 months to reduce this bias and found our results to be consistent with other studies. Finally, our results may be affected by social desirability bias because infection control is a desirable behavior for dental students. Our results may, therefore, underestimate the magnitude of the problem.

## 7. Conclusions

The majority of participants reported a good attitude towards infection control and adequate scores for infection control practice. Nevertheless, an alarming proportion of the students had experienced needle or sharps injury incidents during clinical training without full HBV vaccination coverage. Given the epidemic of blood-borne diseases in Egypt, particularly HBV and HCV, our results suggest that dental students are at an elevated risk of acquiring these during clinical training. Effective and comprehensive infection control education that includes introducing safer products and clinical procedures to minimize the risk of such injuries during clinical practice should be urgently increased in dental education in Egypt. Additionally, refresher training before graduation that focuses on injury prevention and post-exposure protocols is strongly recommended. To develop such education and training programs effectively and efficiently, we suggest further research-especially qualitative studies-to investigate the background of why male or senior students follow precaution protocols less than female or junior students, the context of needle and sharps injuries occurrence, and the reasons behind the lack of complete HBV vaccination coverage among all practicing students from the perspectives of both students and teaching staff.

## Figures and Tables

**Table 1:** Scores of infection control practices among fourth- and fifth-year students in four Egyptian public dental schools.

Infection Control Practice Scores	4th Year				5th Year				*p*-Value ^+^
	Total (*n* = 166)	Male (*n* = 57)	Female (*n* = 109)	*p*-Value **	Total (*n* = 429)	Male (*n* = 115)	Female (*n* = 314)	*p*-Value **	
Self-protection score (range 0–32)	27.9 (2.8) *28.0*	27.0 (2.8) *27.0*	28.3 (2.7) *29.0*	0.005	26.6 (3.2) *27.0*	25.9 (3.8) *26.0*	26.9 (2.9) *27.0*	0.008	<0.001
Missing (*n*)	7	5	2		0	0	0		
Sterilization score (range 0–36)	32.6 (3.0) *33.0*	32.1 (3.1) *33.0*	32.9 (2.9) *33.0*	0.153	31.5 (3.6) *32.0*	30.2 (4.0) *31.0*	31.9 (3.3) *32.0*	<0.001	<0.001
Missing (*n*)	7	5	2		5	2	3		
Injury management score (range 0–12)	8.9 (3.0) *9.0*	8.7 (3.3) *9.5*	9.0 (2.9) *9.0*	<0.001	7.7 (3.0) *8.0*	8.0 (3.1) *8.0*	7.6 (2.9) *8.0*	0.279	<0.001
Missing (*n*)	19	13	6		1	0	1		
Waste management score (range 0–8)	6.7 (1.7) *8.0*	6.9 (1.5) *8.0*	6.7 (1.8) *8.0*	0.526	6.2 (2.0) *7.0*	6.5 (2.0) *7.0*	6.1 (2.0) *6.0*	0.075	0.001
Missing (*n*)	10	5	5		1	1	0		

Mean (standard deviation) and median (italics) calculated excluding missing cases. ** *p*-value from *t*-test comparing mean scores between gender. ^+^ *p*-value from *t*-test comparing mean scores between year totals.

**Table 2:** Scores for risk perception, knowledge, and attitude toward infectious diseases and their control among the third-, fourth-, and fifth-year students in four Egyptian public dental schools.

Risk Perception, Infection Control Attitude and Knowledge Scores	3rd Year				4th Year				5th Year				*p*-Value ^+^
	Total (*n* = 472)	Male (*n* = 157)	Female (*n* = 315)	*p*-Value **	Total (*n* = 166)	Male (*n* = 57)	Female (*n* = 109)	*p*-Value **	Total (*n* = 430)	Male (*n* = 115)	Female (*n* = 314)	*p*-Value **	
Risk perception score (range 0–9)	5.7 (2.3) *6.0*	5.6 (2.2) *6.0*	5.8 (2.3) *6.0*	0.383	5.5 (2.6) *6.0*	5.7 (2.6) *6.0*	5.4 (2.6) *6.0*	0.446	5.0 (2.4) *5.0*	5.0 (2.4) *5.0*	5.0 (2.5) *5.0*	0.947	<0.001
Missing (*n*)	40	9	31		1	0	1		4	0	4		
Attitude toward infection control score (range 0–48)	41.6 (4.4) *43.0*	40.4 (4.3) *41.0*	42.3 (4.3) *43.0*	<0.001	43.3 (3.2) *44.0*	43.0 (3.0) *44.0*	43.5 (3.3) *44.0*	0.402	43.3 (2.8) *44.0*	42.7 (3.4) *43.0*	43.5 (2.5) *44.0*	0.012	<0.001
Missing (*n*)	6	3	3		1	0	1		3	3	0		
Attitude toward patients with infectious diseases score (range 0–9)	3.7 (2.3) *3.0*	4.4 (2.6) *5.0*	3.4 (2.1) *3.0*	<0.001	4.5 (2.5) *4.0*	4.4 (2.2) *4.0*	4.5 (2.6) *4.0*	0.866	3.9 (2.3) *3.0*	4.5 (2.4) *4.0*	3.7 (2.3) *3.0*	0.001	0.003
Missing (*n*)	21	4	17		1	0	1		1	0	1		
Knowledge on infectious diseases score (range 0–6)	1.8 (1.3) *2.0*	1.6 (1.2) *1.0*	1.9 (1.3) *2.0*	0.008	2.0 (1.2) *2.0*	2.0 (1.2) *2.0*	2.0 (1.2) *2.0*	0.651	2.1(1.2) *2.0*	2.2 (1.3) *2.0*	2.0 (1.2) *2.0*	0.202	0.002
Knowledge on infection control score (range 0–11)	4.4 (1.6) *4.0*	4.3 (1.7) *4.0*	4.5 (1.6) *4.0*	0.189	5.1 (1.7) *5.0*	5.3 (1.7) *5.0*	5.0 (1.6) *5.0*	0.324	4.9 (1.5) *5.0*	5.0 (1.5) *5.0*	4.9 (1.5) *5.0*	0.037	<0.001

Mean (standard deviation) and median (italics) calculated excluding missing cases. ** *p*-value from *t*-test comparing mean scores between genders. ^+^ *p*-value from one-way analysis of variance (ANOVA) comparing mean scores between year totals.

**Table 3:** HBV vaccination, needle injury, and sharps injury experiences among the fourth- and fifth-year students in four Egyptian public dental schools.

Hepatitis B Vaccination		4th year				5th year				Total years				*p-*Value *^+^*
		Total *n* = 166 (%)	Male *n* = 57 (%)	Female *n* = 109 (%)	*p-*Value *	Total *n* = 429 (%)	Male *n* = 115 (%)	Female *n* = 314 (%)	*p-*Value *	Total *n* = 595 (%)	Male *n* = 172 (%)	Female *n* = 423 (%)	*p-*Value *	
HBV vaccination status within 6 months	Completed	97 (65.5)	26 (54.2)	71 (71.0)	0.017	293 (72.3)	66 (62.3)	227 (75.9)	0.006	390 (70.5)	92 (59.7)	298 (74.7)	<0.001	0.286
	Incompleted	33 (22.3)	11 (22.9)	22 (22.0)		75 (18.5)	23 (21.7)	52 (17.4)		108 (19.5)	34 (22.1)	74 (18.5)		
	Not vaccinated	18 (12.2)	11 (22.9)	7(7.0)		37 (9.1)	17 (16.0)	20 (6.7)		55 (9.9)	28 (18.18)	27 (6.8)		
	Missing (*n*)	18	9	9		24	9	15		42	18	24		
Experiencing needle injury during clinical practice within the last 6 months	Never	88 (56.4)	38 (73.1)	50 (48.1)	0.012	41 (35.7)	105 (33.8)	146 (34.3)	0.94	234 (40.2)	79 (47.3)	155 (37.3)	0.084	<0.001
	1–3 times	60 (38.5)	12 (23.1)	48 (46.2)		62 (53.9)	173 (55.6)	235 (55.2)		295 (50.7)	74 (44.3)	221 (53.3)		
	>3 times	8(5.1)	2 (3.8)	6(5.8)		12 (10.4)	33 (10.6)	45 (10.6)		53(9.1)	14(8.4)	39(9.4)		
	Missing (*n*)	10	5	5		3	0	3		13	5	8		
Experiencing sharps injury during clinical practice within the last 6 months	Never	113 (72.9)	42 (80.8)	71 (68.9)	0.131	244 (57.7)	76 (66.1)	168 (54.5)	0.08	357 (61.8)	118 (70.7)	239 (58.2)	0.020	0.004
	1–3 times	35 (22.6)	7 (13.5)	28 (27.2)		149 (35.2)	34 (29.6)	115 (37.3)		184 (31.8)	41 (24.6)	143 (34.8)		
	>3 times	7 (4.5)	3 (5.8)	4 (3.9)		30 (7.1)	5 (4.3)	25 (8.1)		37 (6.4)	8 (4.8)	29 (7.1)		
	Missing (*n*)	11	5	6		6	0	6		17	5	12		

* *p*-value from X^2^-test comparing the distribution of HBV vaccination status or injury experience between genders of the same year. ^+^ *p*-value from X^2^-test comparing the distribution of HBV vaccination status or injury experience between year totals. *p*-values from likelihood ratio tests were used for the comparison between years when >20% of expected cell counts were less than 5.

**Table 4:** Bivariate and multivariate logistic analyses for correlates of infection control practices among students in four Egyptian public dental schools.

		Self-Protection (*n* = 588)				Sterilization (*n* = 583)				Injury Management (*n* = 575)				Waste Management (*n* = 584)			
		COR (95% CI)	*p-*Value	AOR (95% CI)	*p-*Value	COR (95% CI)	*p-*Value	AOR (95% CI)	*p-*Value	COR (95% CI)	*p-*Value	AOR (95% CI)	*p-*Value	COR (95% CI)	*p-*Value	AOR (95% CI)	*p-*Value
Year grade	Year 4 (ref: Year 5)	2.2 (1.5–3.2)	<0.001	2.6 (1.6–4.1)	<0.001	2.2 (1.4–3.2)	<0.001	2.3 (1.4–3.7)	0.001	2.2 (1.5–3.4)	<0.001	1.7 (1.0–2.7)	0.036	1.7 (1.2–2.5)	0.005	1.9 (1.2–2.9)	0.005
Gender	Female (ref: Male)	1.6 (1.2–2.3)	0.014	1.7 (1.1–2.4)	0.011	1.8 (1.3–2.6)	0.001	1.8 (1.2–2.8)	0.003	0.9 (0.6–1.3)	0.493			0.7 (0.5–1.0)	0.037	0.6 (0.4–0.9)	0.024
HBV vaccination course within 6 months	Complete (ref: Not vaccinated)	1.3 (0.8–2.3)	0.323			1.5 (0.9–2.7)	0.161			1.0 (0.6–1.9)	0.914			1.4 (0.8–2.4)	0.264		
	Incomplete (ref: Not vaccinated)	1.2 (0.6–2.3)	0.57			1.1 (0.6–2.2)	0.728			1.1 (0.5–2.1)	0.855			1.1 (0.6–2.2)	0.696		
	Missing	1.5 (0.6–3.6)	0.346			1.1 (0.5–2.7)	0.782			1.7 (0.7–4.3)	0.256			1.8 (0.7–4.2)	0.203		
Experiencing needle injury during clinical practice within the last 6 months	Never (ref: >3 times)	2.1 (1.2–3.9)	0.015	1.6 (0.8–3)	0.160	3.1 (1.7–5.8)	<0.001	2.3 (1.1–4.7)	0.030	3.1 (1.8–6.1)	<0.001	2.4 (1.2–4.9)	0.012	1.5 (0.8–2.8)	0.164		
	1–3 times (ref: >3 times)	1.6 (1.0–2.9)	0.108	1.3 (0.7–2.5)	0.378	2.7 (1.5–4.9)	0.001	2.1 (1.0–4.3)	0.045	1.5 (0.8–2.8)	0.161	1.4 (0.7–2.7)	0.388	1.1 (0.6–1.9)	0.828		
	Missing	2.4 (0.4–14.4)	0.332	1.4 (0.2–10.0)	0.724	1.6 (0.3–8.7)	0.587	1.0 (0.1–8.7)	0.994	5.2 (0.5–49.9)	0.152	4.1 (0.3–52.2)	0.279	0.6 (0.1–4.2)	0.642		
Experiencing sharps injury during clinical practice within the last 6 months	Never (ref: >3 times)	1.3 (0.6–2.5)	0.475			2.5 (1.2–4.9)	0.010	1.2 (0.5–2.8)	0.632	2.0 (1.0–4.0)	0.047	1.3 (0.6–2.9)	0.464	1.0 (0.5–2.0)	0.945		
	1–3 times (ref: >3 times)	0.8 (0.4–1.6)	0.491			1.9 (0.9–3.8)	0.085	1.1 (0.5–2.5)	0.897	0.8 (0.4–1.6)	0.567	0.6 (0.3–1.4)	0.239	0.8 (0.4–1.7)	0.611		
	Missing	3.0 (0.6–16.4)	0.194			2.0 (0.5–8.2)	0.351	1.5 (0.2–9.3)	0.662	3.3 (0.6–18.1)	0.167	1.7 (0.3–11.6)	0.562	1.5 (0.3–7.0)	0.590		
Risk perception (Median = 6)	≥Median (ref: <Median)	1.1 (0.8–1.5)	0.688			1.4 (1.0–2.0)	0.040	1.6 (1.1–2.3)	0.014	0.8 (0.6–1.4)	0.222			0.9 (0.6–1.3)	0.538		
	Missing	1.1 (0.2–6.6)	0.927			3.0 (0.3–27.1)	0.331	3.4 (0.4–32.7)	0.286	0.4 (0.1–2.4)	0.313			0.5 (0.1–3.0)	0.448		
Attitude toward patients with infectious diseases (Median = 4)	≥Median (ref: <Median)	1.3 (0.9–1.8)	0.132	1.3 (0.9–1.9)	0.143	1.0 (0.7–1.4)	0.986			1.4 (1.0–1.9)	0.069	1.4 (1.0–2.1)	0.047	1.3 (1.0–1.8)	0.096	1.3 (0.9–1.9)	0.107
Attitude toward infection control (Median = 44)	≥Median (ref: <Median)	1.9 (1.4–2.7)	<0.001	1.7 (1.2–2.5)	0.003	3.1 (2.2–4.4)	<0.001	2.7 (1.9–3.9)	<0.001	1.9 (1.3–2.6)	<0.001	1.8 (1.2–2.6)	0.002	2.2 (1.6–3.1)	<0.001	2.3 (1.6–3.3)	<0.001
Knowledge on infectious diseases (Median = 2)	≥ Median (ref: <Median)	1.1 (0.8–1.5)	0.684			1.4 (1.0–2.0)	0.035	1.4 (10–2.0)	0.096	0.8 (0.6–5.35)	0.259			1.0 (0.7–1.4)	0.849		
Knowledge on infection control (Median = 5)	≥Median (ref: Median)	1.0 (0.7–1.4)	0.878			1.1 (0.8–1.6)	0.552			0.9 (0.7–1.3)	0.728			1.0 (0.7–1.4)	0.856		

—COR: crude odds ratio; AOR: adjusted odds ratio; CI: confidence interval. The model was adjusted for universities.—Odds ratios for missing values for year, gender, attitude toward patients with infectious diseases, attitude toward infection control, knowledge of infectious diseases, or knowledge of infection control were not included in the analysis because numbers of missing values were <5.

## Supplementary Materials

The following are available online at https://www.mdpi.com/article/10.3390/ijerph18126248/s1, Questionnaire S1, Data S1, Table S1. Needle injury and sharp injury experiences among the groups of 4th year and 5th year students with different HBV vaccination experience in 4 Egyptian public dental schools.
