# Supplementary material for: Infection Control Knowledge, Attitudes, and Practices among Students of Public Dental Schools in Egypt
Source: Int J Environ Res Public Health. 2021 Jun 9;18(12):6248. doi: 10.3390/ijerph18126248 (PMC8296034; doi:10.3390/ijerph18126248)
Supplement: Supplementary file 1 [file ijerph-18-06248-s001.zip › ijerph-1155629-JP-supplementary/Questionanire S1.pdf]

Infection control Knowledge, Attitude and Practice among Undergraduate students in Public Dental Schools, Egypt

Level 3 4 5

Gender Male Female

Age ………………………

Have you studied all university levels in this university so far? 1- Yes Inside Egypt Outside Egypt
2- No

Practice (This section was not included for pre-clinical (third) level)

1-Self-protection

No.	Practice	Always	Often	sometimes	rarely	never
1.1	I wash my hands with water and soap before and after treating each patient					
1.2	I use alcohol rub before and after treating each patient					
1.3	I use gloves when treating patients					
1.4	I use new gloves for treating each patient					
1.5	I use mask when treating patients					
1.6	I use new mask for treating each patient					
1.7	I use eye protection (eye goggles/face shield) when treating patients					
1.8	I use a special gown /apron when treating patients					
1.9	Did you get vaccinated for Hepatitis virus?	**1- Yes** → 1.9.1- how many shots have you taken within 6 months? 1- One shot; 2- Two shots; 3- Three shots; 4- I am currently taking the shots<br>**2- No**				

2- *Instruments sterilization and environment disinfection*

No.	Practice	Always	Often	sometimes	rarely	never
**1.12**	I disinfect impressions before sending them to the technician	☐	☐	☐	☐	☐
**1.13**	I disinfect extracted teeth which i use for training (example for endodontic labs)	☐	☐	☐	☐	☐
**1.14**	I disinfect treating surfaces (like tray,chair,light) before treating each patient	☐	☐	☐	☐	☐
**1.15**	I dispose saliva ejector after treating each patient	☐	☐	☐	☐	☐
**1.16**	I use new rinsing cup for each patient	☐	☐	☐	☐	☐
**1.17**	I use sterilized burs for each patient	☐	☐	☐	☐	☐
**1.18**	I use sterilized endodontic files for each patient	☐	☐	☐	☐	☐
**1.19**	I use new anesthesia needle for more than one patient	☐	☐	☐	☐	☐
**1.20**	I use new anesthesia cartridge (carpule) for more than one patient	☐	☐	☐	☐	☐

3-Injury exposure and waste management

2.1– How often do you recall getting injured by needle stick during treating patients within the last 6 months?

1- Always (more than 6 times) ☐
2- Often (4-5 times) ☐
3- Sometimes (2-3 times) ☐
4- Rarely (one time) ☐
5- Never ☐

2.2- How often do you recall getting injured by sharp instrument during treating patients within the last 6 months?

1- Always (more than 6 times) ☐
2- Often (4-5 times) ☐
3- Sometimes (2-3 times) ☐
4- Rarely (one time) ☐
5- Never ☐

No.	Practice	Always	Often	sometimes	rarely	never
2.3	I would inform my clinical supervisor if i get injured by sharps during treating patients	☐	☐	☐	☐	☐
2.4	I would wash my hands immediately if i get injured by sharps in the clinic even if there is no visible bleeding	☐	☐	☐	☐	☐
2.5	I would do blood test if i get injured during treating patients	☐	☐	☐	☐	☐
2.6	I dispose needles and disposable sharps (eg. scalpel tips) in a special container other than the regular trash	☐	☐	☐	☐	☐
2.7	I dispose biological waste (eg.extracted teeth) in a special container other than the regular trash	☐	☐	☐	☐	☐

Attitude

Risk perception

No.	*Question*	*Highly expected*	*expected*	*Not very much expected*	*Never expected*
3.1	I am susceptible to **HBV** because of my clinical practice	☐	☐	☐	☐
3.2	I am susceptible to **HCV** because of my clinical practice	☐	☐	☐	☐
3.3	I am susceptible to **HIV** because of my clinical practice	☐	☐	☐	☐

Attitude toward treating patients with infectious diseases

No.	Question	I treat	I treat with some concern	A lot of concern but I still treat	I will not treat	*If the answer is "I will not treat" Why?*
3.5	Treating patient who has HBV	☐	☐	☐	☐ →	3.5.1<br>1- To avoid getting infected ☐<br>2-Afraid if my peers and patients know about it ☐<br>3- I do not have enough knowledge about dealing with patients with HBV infections to avoid trans infection ☐

**3.6**	Treating patient who has HCV	☐	☐	☐	☐ →	3.6.1<br>1- To avoid getting infected ☐<br>2-Afraid if my peers and patients know about it ☐<br>3- I do not have enough knowledge about dealing with patients with HCV infections to avoid trans infection ☐
**3.7**	Treating patient who has HIV/AIDS	☐	☐	☐	☐ →	3.7.1<br>1- To avoid getting infected ☐<br>2-Afraid if my peers and patients know about it ☐<br>3- I do not have enough knowledge about dealing with patients with HIV infections to avoid trans infection ☐

Attitude toward infection control measures

No.	***Question***	***Strongly agree***	***Agree***	***Neutral***	***Somehow disagree***	***Absolutely disagree***
4.1	It is important to use gloves when treating patients	☐	☐	☐	☐	☐
4.2	*It is important to use new gloves for treating each patient*	☐	☐	☐	☐	☐
4.3	*It is important to use alcohol rub after treating each patient*	☐	☐	☐	☐	☐
4.4	*It is important to use mask when treating patients*	☐	☐	☐	☐	☐
4.5	*It is important to use new mask for treating each patient*	☐	☐	☐	☐	☐

4.6	*It is important to get HBV vaccine before starting clinical practice*	☐	☐	☐	☐	☐
4.7	*Re-usable instruments should be sterilized for each patient*	☐	☐	☐	☐	☐
4.8	*Treatment surfaces should be disinfected after each treatment*	☐	☐	☐	☐	☐
4.9	*Disposable tools (eg. saliva ejector) should be changed for each patient*	☐	☐	☐	☐	☐
4.10	*It is important to dispose sharps in a special container*	☐	☐	☐	☐	☐
4.11	*It is not important to use new anesthesia needles for each patient (recode SPSS)*	☐	☐	☐	☐	☐
4.12	*It is not important to use new anesthesia cartridge (carpule) for each patient (recode SPSS)*	☐	☐	☐	☐	☐

Knowledge 1- on Infectious diseases **(Multiple answers can be selected)**

No.	Question
**5.1**	**HBV virus is transmitted via:**<br>1- ☐ Blood<br>2- ☐ Unprotected sex<br>3- ☐ Saliva<br>4- ☐ All of the above<br>5- ☐ None of the above<br>6- ☐ I do not know
**5.2**	**HBV transmission can be prevented by:**<br>1- ☐ Vaccine<br>2- ☐ Avoid direct contact with patient's fluids<br>3- ☐ Avoid direct contact with the patient's blood<br>4- ☐ Avoid any contact with the patient<br>5- ☐ All of the above<br>6- ☐ I do not know

**5.3**	**HCV virus is transmitted via:**<br>1- ☐ Blood<br>2- ☐ Unprotected sex<br>3- ☐ Saliva<br>4- ☐ All of the above<br>5- ☐ None of the above<br>6- ☐ I do not know
**5.4**	**HCV transmission can be prevented by:**<br>1- ☐ Vaccine<br>2- ☐ Avoid direct contact with patient's fluids<br>3- ☐ Avoid direct contact with the patient's blood<br>4- ☐ Avoid any contact with the patient<br>5- ☐ All of the above<br>6- ☐ I do not know
**5.5**	**HIV virus is transmitted via:**<br>1- ☐ Blood<br>2- ☐ Unprotected sex<br>3- ☐ Saliva<br>4- ☐ All of the above<br>5- ☐ None of the above<br>6- ☐ I do not know
**5.6**	**HIV transmission can be prevented by:**<br>1- ☐ Vaccine<br>2- ☐ Avoid direct contact with patient's fluids<br>3- ☐ Avoid direct contact with the patient's blood<br>4- ☐ Avoid any contact with the patient<br>5- ☐ All of the above<br>6- ☐ I do not know

2- Knowledge on infection control measures (one answer to be selected)

**6.1**	The most important measure to decrease the transmissibility of diseases in health facilities is:<br>1- ☐ Using gloves<br>2- ☐ Hand disinfection<br>3- ☐ Instruments sterilization<br>4- ☐ I do not know
**6.2**	We can prevent hand from infection when touching infected saliva, blood or mucous by:<br>1- ☐ Using gloves<br>2- ☐ Hand disinfection<br>3- ☐ Instruments sterilization<br>4- ☐ I do not know
**6.3**	The best method to disinfect hands is:<br>1- ☐ Using alcohol-based rub<br>2- ☐ Washing hands with soap and water<br>3- ☐ Washing hands with antimicrobial soap<br>4- ☐ I do not know

**6.4**	The most appropriate autoclave type for dental instruments sterilization is:<br>1- ☐ S-type<br>2- ☐ B-type<br>3- ☐ N-type<br>4- ☐ I do not know
**6.5**	The importance of colored indicators on the sterilization bags is:<br>1- ☐ To indicate expiratory date<br>2- ☐ To indicate the proper temperature and pressure for sterilization<br>3- ☐ none of the above<br>4- ☐ I do not know
**6.6**	The most Suitable temperature for dry heat (oven) sterilization:<br>1- ☐ 200C<br>2- ☐ 100C<br>3- ☐ 160C<br>4- ☐ I do not know
**6.7**	An example of intermediate level disinfectant is:<br>1- ☐ Glutaraldehyde<br>2- ☐ Ethyl alcohol solutions<br>3- ☐ Dettol<br>1- ☐ I do not know
**6.8**	Among the following is **most crucially** to be discarded in a special container:<br>1- ☐ Used cotton<br>2- ☐ Used needles<br>3- ☐ None of the above<br>4- ☐ I do not know
**6.9**	The importance of using mask and protective face shields is to protect:<br>1- ☐ Exposed part of the skin (exposed due to injury for example)<br>2- ☐ Un exposed part of the skin<br>3- ☐ All of the above<br>4- ☐ I do not know
**6.10**	The disease with highest potentiality for transmission due to needle injury is:<br>1- ☐ HBV<br>2- ☐ HCV<br>3- ☐ HIV<br>4- ☐ I do not know
**6.11**	The infectious disease with available vaccination is:<br>1- ☐ HBV<br>2- ☐ HCV<br>3- ☐ HIV<br>4- ☐ I do not know
