# Supplementary material for: Infection Control Knowledge, Attitudes, and Practices among Students of Public Dental Schools in Egypt
Source: Int J Environ Res Public Health. 2021 Jun 9;18(12):6248. doi: 10.3390/ijerph18126248 (PMC8296034; doi:10.3390/ijerph18126248)
Supplement: Supplementary file 1 [file ijerph-18-06248-s001.zip › ijerph-1155629-JP-supplementary/Table S1.pdf]

Table S1. Needle injury and sharp injury experiences among the groups of 4th year and 5th year students with different HBV vaccination experience in 4 Egyptian public dental schools.

		**4th Year**								**5th Year**								**Total of All Years**			***p*-Value** ⁺
		**Male** ***n*** **(%)**		**Female** ***n*** **(%)**		**Total** ***n*** **(%)**			***p*-Value** *	**Male** ***n*** **(%)**		**Female** ***n*** **(%)**		**Total** ***n*** **(%)**			***p*-Value** *				
		NC (*n* = 22)	C (*n* = 26)	NC (*n* = 29)	C (*n* = 71)	NC (*n* = 51)	C (*n* = 97)	Total (*n* = 148)		**NC (*n* = 40)**	**C (*n* = 66)**	**NC (*n* = 72)**	**C (*n* = 227)**	**NC (*n* = 112)**	**C (*n* = 293)**	**Total (*n* = 405)**		**NC (*n* = 163)**	**C (*n* = 390)**	**Total (*n* = 553)**	
Got injured by needle during clinical practice within the last 6 months	Never	18 (81.8)	19(73.1)	16 (55.2)	30 (44.1)	34(66.7)	49 (52.1)	83 (57.2)		17 (42.5)	20 (30.3)	23 (32.9)	78 (34.4)	40 (36.4)	98 (33.4)	138 (34.2)		74 (46.0)	147 (38.0)	221 (40.3)	
	1–3 times	4 (18.2)	6 (23.1)	12 (41.4)	34 (50.0)	16 (31.4)	40 (42.6)	56 (38.6)		17 (42.5)	42 (63.6)	38 (54.3)	129 (56.8	55 (50.0)	171 (58.4)	226 (56.1)		71 (44.1)	211 (54.5)	282 (51.5)	
	>3 times	0 (0.0)	1 (3.8)	1 (3.4)	4 (5.9)	1 (2.0)	5 (5.3)	6 (4.1)	0.190	6 (15.0)	4 (6.1)	9 (12.9)	20 (8.8)	15 (13.6)	24 (8.2)	39 (9.7)	0.162	16 (9.9)	29 (7.5)	45 (8.2)	0.081
	Missing	0	0	0	3	0	3	3		0	0	2	0	2	0	2		2	3	5	
Got injured by sharps during clinical practice within the last 6 months	Never	20 (90.9)	20 (76.9)	17 (60.7)	48 (70.6)	37 (74.0)	68 (72.3)	105 (72.9)		28 (70.0)	43 (65.2)	38 (54.3)	121 (54.0)	66 (60.0)	164 (56.6)	230 (57.5)		103 (64.4)	232 (60.4)	335 (61.6)	
	1–3 times	1 (4.5)	5 (19.2)	9 (32.1)	18 (26.5	10 (20.0)	23 (24.5)	33 (22.9)	0.641	10 (25.0)	21 (31.8)	24 (34.3)	86 (38.4)	34 (30.9)	107 (36.9)	141 (35.3)	0.431	44 (27.5)	130 (33.9)	174 (32.0)	0.257
	>3 times	1 (4.5)	1 (3.8)	2 (7.1)	2 (2.9)	3 (6.0)	3 (3.2)	6 (4.2)		2 (5.0)	2 (3.0)	8 (11.4)	17 (7.6)	10 (9.1)	19 (6.6)	29 (7.2)		13 (8.1)	22 (5.7)	35 (6.4)	
	Missing	0	0	1	3	1	3	4		0	0	2	3	2	3	5		3	6	9	

NC = No vaccination/incomplete vaccination course within the past 6 months. C = Complete vaccination course within the past 6 months. * *p*-value from X^2-test comparing needle and injury status between students with NC and C within the same year grade regardless of gender. ⁺ *p*-value from X^2-test comparing needle and injury status between total students with NC and C regardless of gender.
